# Correction

**DOI:** 10.1080/20008066.2024.2355062

**Published:** 2024-06-14

## Effects of intensive trauma-focused treatment of individuals with both post-traumatic stress disorder and borderline personality disorder

K. A. Kolthof, E. M. Voorendonk, A. Van Minnen & A. De Jongh

*European Journal of Psychotraumatology*, 13:2, 2143076, 2022

https://doi.org/10.1080/20008066.2022.2143076

During preparation for another study, coding errors were discovered. These errors involved mistakenly coding some female participants as male. Further investigation showed that this coding error also affected some of the data used in this study. Although quite a number of women were coded as male (*n* = 12), this did not change the outcomes of the study, but it did change the significance of some of the results. Therefore, we would like to correct the content of this article. All corrections involve (only) the Results section:

## Correction 1.

3.1. Patient flow and sample characteristics. The baseline sample consisted of 45 patients. Of this group, 60% (*n* = 27) were female with an average age of 41.10 years (SD = 12.74).

Now reads:

3.1. Patient flow and sample characteristics. The baseline sample consisted of 45 patients. Of this group, 87% (*n* = 39) were female with an average age of 41.10 years (SD = 12.74).

## Correction 2.

3.2. Change in PTSD symptom severity and diagnostic status at post-treatment, 6-months, and 12-month follow-up (CAPS-5). …  … A significant interaction effect between time and gender was found, reflecting a significantly steeper CAPS-5 decline in male participants than in their female counterparts (F[2,70] = 6.263, *p* = .003).

Now reads:

3.2. Change in PTSD symptom severity and diagnostic status at post-treatment, 6-months, and 12-month follow-up (CAPS-5). …  … No significant interaction effect between time and gender was found (F[2,70] = 1.379, *p* = .259).

## Correction 3.

3.3. Change in BPD symptom severity and diagnostic status at post-treatment, 6-month, and 12-month follow-up (BPDSI-IV and SCID-5- P … ..No significant time by gender interaction regarding the BPDSI-IV score was found (F[1.67, 58.39] = 2.91, *p* = .071).

Now reads:

3.3. Change in BPD symptom severity and diagnostic status at post-treatment, 6-month, and 12-month follow-up (BPDSI-IV and SCID-5- P … ..No significant time by gender interaction regarding the BPDSI-IV score was found (F[1.63, 57.01]  = , *p* = 0.225).

Table 1 has been recalculated. Changes as compared to the published article are highlighted.

**Table 1.** Baseline characteristics of patients with posttraumatic stress disorder and a comorbid borderline personality disorder.

Variable	Total group *N* = 45 N (%)
Gender female (%)	39 (87.0)
Trauma exposure	
Sexual abuse	42 (93.3)
Sexual abuse < 12 years	32 (71.1)
Sexual abuse > 12 years	10 (22.2)
Physical abuse such as being attacked, punched of kicked	42 (93.3)
Witnessed sudden violent death such as murder of suicide	24 (53.3)
Comorbidity Depression	29 (64.4)
Specific phobia	6 (13.3)
Generalised anxiety disorder	3 (6.7)
Obsessive compulsive disorder	8 (17.8)
Panic disorder	8 (17.8)
Agoraphobia	14 (31.1)
Social anxiety disorder	14 (31.1)
Alcohol dependency or abuse	8 (17.8)
Eating disorder	4 (8.9)
Bipolar disorder	4 (8.9)
Substance dependency or abuse	10 (22.2)
(hypo)mania	8 (17.8)
Psychotic disorders	13 (28.9)
	M (SD)
Age	41.10 (12.74)

